# Thrombosed capillary network in a full‐thickness burn

**DOI:** 10.1002/ccr3.3048

**Published:** 2020-06-22

**Authors:** Sharon Kennedy, Kevin C. Cahill

**Affiliations:** ^1^ National Burns Unit Department of Plastic and Reconstructive Surgery St. James's Hospital Dublin 8 Ireland

**Keywords:** critical care medicine, emergency medicine, general surgery

## Abstract

Full‐thickness burns are associated with cardinal clinical features. The recognition of these signs has important implications for immediate and future care of such burns.

## Graphical abstract

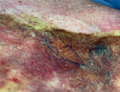

An 83‐year‐old man sustained a 20% total body surface area scald burn to his anterior chest, bilateral upper limbs and upper back following a fall at home during which he inadvertently pulled a pot of boiling water on to himself. He sustained no other injuries in the fall. He received minimal first aid but was rapidly transferred to a National Burns Unit. His burn was assessed. Thrombosed capillary networks were seen indicating full‐thickness burns (Figure 1). In full‐thickness burns, the epidermis, dermis, adnexal structures, and to a varying degree, the subcutaneous tissue is destroyed by thermal insult, and in this case, the subdermal capillary network is involved. Full‐thickness burns are characterized by eschar, lack of capillary refill, and lack of cutaneous sensation.^1^ With thrombosed burnt capillaries, it is intuitive that capillary refill will be absent in the area supplied by those vessels. The patient underwent tangential burns excision and autografting. The course of his hospital admission was complicated by pneumonia, and he died secondary to respiratory sepsis eight days following his admission.

**FIGURE 1:**
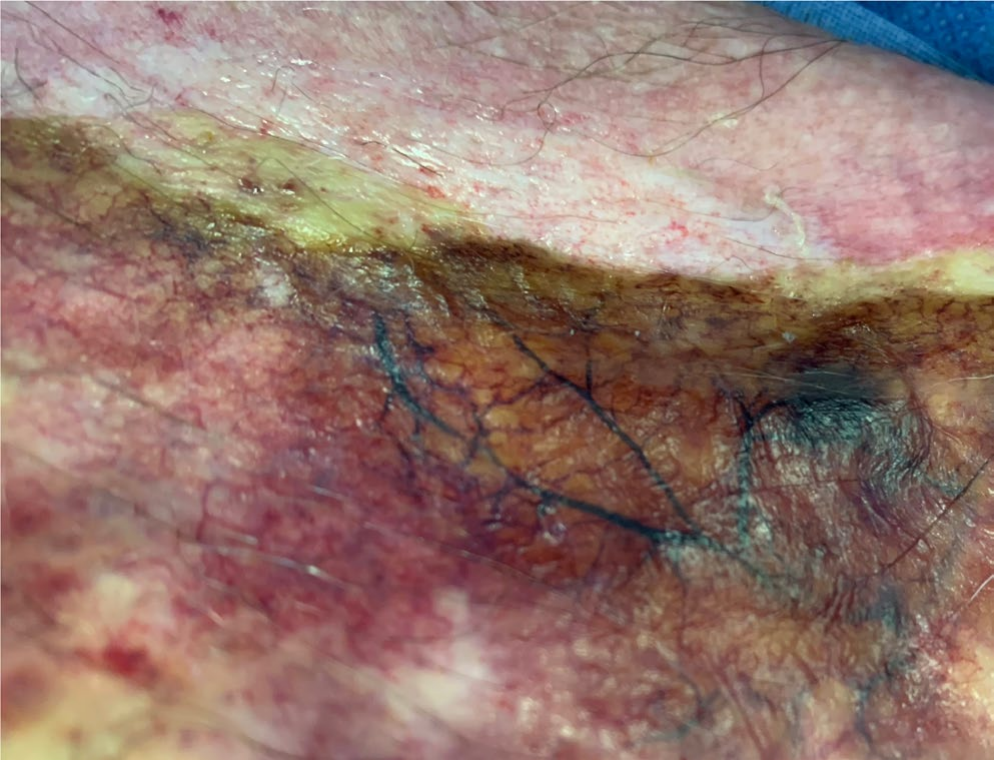
Thrombosed capillary network within a full‐thickness burn

## CONFLICT OF INTEREST

None declared.

## AUTHOR CONTRIBUTIONS

SK: drafted and reviewed the article. KCC: reviewed the article.
